# Host-specific signatures of the cell wall changes induced by the plant parasitic nematode, *Meloidogyne incognita*

**DOI:** 10.1038/s41598-018-35529-7

**Published:** 2018-11-23

**Authors:** Refik Bozbuga, Catherine J. Lilley, J. Paul Knox, Peter E. Urwin

**Affiliations:** Centre for Plant Sciences, Faculty of Biological Sciences, University of Leeds, LS2 9JT Leeds, United Kingdom

## Abstract

Root-knot nematodes (*Meloidogyne* spp.) are an important group of plant parasitic nematodes that induce within host plant roots unique feeding site structures, termed giant cells, which supply nutrient flow to the nematode. A comparative *in situ* analysis of cell wall polysaccharides in the giant cells of three host species (Arabidopsis, maize and aduki bean) infected with *Meloidogyne incognita* has been carried out. Features common to giant cell walls of all three species include the presence of high-esterified pectic homogalacturonan, xyloglucan and pectic arabinan. The species-specific presence of xylan and mixed-linkage glucan (MLG) epitopes in giant cell walls of maize reflected that host’s taxonomic group. The LM5 galactan and LM21 mannan epitopes were not detected in the giant cell walls of aduki bean but were detected in Arabidopsis and maize giant cell walls. The LM2 arabinogalactan-protein epitope was notable for its apparent global variations in root cell walls as a response to infection across the three host species. Additionally, a set of Arabidopsis cell wall mutants were used to determine any impacts of altered cell wall structures on *M*. *incognita* infection. Disruption of the *arabinogalactan-protein 8* gene had the greatest impact and resulted in an increased infection rate.

## Introduction

Plant parasitic nematodes are obligate parasites that infect mainly root tissue of a wide range of plant species. They can be classified as sedentary or migratory depending on their association with the host plant. Sedentary endoparasitic nematodes have the most complex interactions with their host. They invade roots soon after hatching and then establish a permanent feeding site from which nutrients are withdrawn for the remainder of the nematode’s life. A large proportion of nematode damage to crops worldwide is inflicted by two major groups of sedentary endoparasites, the cyst nematodes (*Heterodera* spp. and *Globodera* spp.) and root-knot nematodes (*Meloidogyne* spp; RKN) that induce specialised feeding structures termed syncytia and giant cells respectively^1,2^. Although these two types of feeding site share some structural features and a common function as a sink tissue for delivering nutrients to the nematode, they are formed by distinct processes^3^.

Root-knot nematodes are considered the most economically important plant parasitic nematodes^4^ as the various *Meloidogyne* spp. are between them capable of infecting almost all species of higher plants^5^. These endoparasites spend most of their life cycle within plant roots. The motile second stage juveniles (J2s) penetrate behind the root tip, usually in the zone of elongation, and migrate intercellularly towards the apical meristematic region. There they turn around and migrate back away from the root tip until they reach the differentiating vascular tissue where they induce feeding site formation. The nematode initiates the development of the feeding site by piercing cell walls with its stylet, through which pharyngeal gland secretions are released. The formation of the feeding site involves re-differentiation of a small number of cells into multinucleate, hypertrophied feeding cells known as giant cells, which reach a maximum size within two weeks. Their expansion is associated with increases in cell wall thickness and the density and volume of cytoplasm, proliferation of endoplasmic reticulum, ribosomes, mitochondria, and plastids and the replacement of the large central vacuole with numerous small vacuoles^2,6^. The wall of giant cells has an irregular surface^6^. Cell wall ingrowths proliferate as root-knot nematodes develop, then degenerate as nematodes reach maturity and complete their life cycle. These wall ingrowths, which are particularly prominent adjacent to xylem vessels, notably increase the surface area of the plasma membrane, assisting the transport of nutrients into or out of the feeding cell^7^. The cells surrounding the giant cells undergo proliferation and enlargement resulting in the formation of the typical gall structure^7^.

Plant cell walls have fundamental roles that include cell and organ growth, defence, intercellular communication and tissue/organ mechanical properties^8,9^. Cell walls can be divided into the primary walls of growing cells and the secondary walls (in certain cells only) which are thickened structures deposited after cell expansion has ceased. Both primary and secondary cell walls are constituted of cellulose, matrix polysaccharides and structural proteins and in some cases secondary cell walls can be lignified^10^. Matrix polysaccharides that are co-extensive with cellulose microfibrils are combinations of xyloglucans, heteroxylans, heteromannans and the complex pectic group of polysaccharides that includes homogalacturonan (HG) and the hypervariable rhamnogalacturonan-I^11–14^. In addition, sets of glycoproteins such as extensins and arabinogalactan-proteins (AGPs) can contribute to structural and/or signalling features of plant cell surfaces^15,16^. There is a broad division amongst angiosperms in relation to cell wall matrix polysaccharide biochemistry. Eudicots and non-commelinid monocots have a primary cell wall matrix dominated by xyloglucan and pectic polysaccharides. In the commelinid monocotyledons, matrix polysaccharides are predominantly glucuronoarabinoxylans with relatively lower levels of xyloglucan and pectins^17^. Moreover, an additional feature of grass cell walls, absent from those of other angiosperms, is the presence of a mixed-linkage glucan^17^. In order to understand in detail the structures and formation of the cell walls of giant cells a panel of monoclonal antibodies was used to elucidate the major wall components in giant cells induced by RKN in three different plant species that encompass both grass (*Zea mays*) and eudicot hosts (*Arabidopsis thaliana*, *Vigna angularis*). In addition a series of Arabidopsis mutations influencing cell wall structures were studied in relation to their impact on RKN infection.

## Materials and Methods

### Plant materials and growth

*Arabidopsis thaliana* (L) Heynh, ecotype Columbia-0 (Col-0) seeds were sterilised by incubation for 30 s in 70% ethanol followed by a 30 min incubation in commercial bleach (10%). After five washes with sterile tap water the seeds were placed overnight at 4 °C. Two sterilised seeds were sown on each 100 mm square Petri dish (Sterilin Ltd., Newport, United Kingdom) containing solidified Gamborg’s B5 medium including vitamins (Duchefa Biochemie, Haarlem, the Netherlands) with 15 g/l sucrose and 10 g/l plant agar (pH 5.5–5.8). Plates were placed in a Sanyo environmental test chamber at 25 °C under 16 h light; 8 h dark cycles with 30% humidity and light intensity was 140 µmol m^−2^s^−1^. Roots were inoculated with nematodes when the plants were 15 days old.

Seeds of aduki bean (*Vigna angularis*) and maize (*Zea mays*) were placed into the upper fold of growth pouches (Mega International) that were held vertically in a deep plastic box filled with tap water to a depth of approximately 2 cm. The pouches were placed in the growth conditions described above for Arabidopsis. Root systems were inoculated with nematodes after 14 days of growth.

### Nematode culture and infection

*Meloidogyne incognita* population VW6 was maintained on tomato plants (‘Ailsa Craig’) growing in potting compost in a glasshouse at 25 °C. For extraction of infective 2^nd^-stage juveniles (J2s) of *M*. *incognita*, galled tomato plant roots with visible egg masses (about two months post infection) were washed, cut into 3–4 cm lengths and placed in a mist chamber at 25 °C. Collected J2s were pelleted in 1.5 ml microcentrifuge tubes (Axygen, Maxymum Recovery) at 3000 rpm for 30 s and sterilised, if necessary, in 0.1% chlorhexidine digluconate and 0.5 mg/ml hexadecyltrimethylammonium bromide (CTAB) for 30 min at room temperature on a rotational mixer. Additional sterilisation involved successive 5 min incubations in streptomycin (1 mg/ml) and penicillin (1000 units/ml), amphotericin B (50 µg/ml) and CTAB. Following three washes with sterile 0.01% Tween-20, J2 numbers were adjusted to 1 nematode per 1 µl for infection of Arabidopsis plant roots in sterile tissue culture.

Unsterilized J2 of *M*. *incognita* were used to infect roots of aduki bean and maize in pouches. In all cases, five root tips per plant were inoculated with 20 J2 nematodes per tip and covered by small (<1 cm^2^) pieces of Glass Microfibre Filter (GF/A – Whatman) paper. Uninfected plants were prepared as controls. After 48 h of nematode infection, GF/A papers were removed in order to achieve synchronous nematode infection. Root samples were collected at 21 days post infection (dpi) and root segments harbouring single galls were identified, cut by scalpel and collected into sterile water in a 1.5 ml microcentrifuge tube. Uninfected root segments of the same size, age and relative location were collected similarly from control plants.

### *In situ* cell wall analysis

#### Sample preparation

Root segments were fixed overnight at 4 °C in 4% paraformaldehyde in PEM buffer (50 mM PIPES, 5 mM EGTA, 5 mM MgSO_4_; pH 6.9) then washed three times in 1x phosphate-buffered saline (PBS; Sigma Chemical, UK). Roots were dehydrated in an ethanol series as follows; 10%, 20%, 30%, 50% ethanol incubations each for 30 min and 70%, 90%, 100% ethanol incubations each for 60 min. After dehydration, the samples were infiltrated with a dilution series of LR White resin (R1280, hard grade; Agar Scientific) in ethanol (10%, 20%, 30%, 50% for 30 min each; 70%, 90%, 100% for 60 min each). The root samples were then maintained in 100% resin overnight at 4 °C for 2 days with 3 changes of resin. Individual root samples were placed into gelatine capsules (G29204; Agar Scientific; Size 4, Essex, UK) filled with 100% resin and incubated at 37 °C for five days. A microtome (Ultracut, Reichert-Jung) with a diamond blade (G339-10, Diatome histoknife, 6.0 mm S/N, Agar Scientific) was used to collect 0.5 µm thick transverse root sections that were mounted onto Vectabond^TM^ (Vector Laboratories) treated multi-well slides (MP Biomedicals).

#### Immunolabelling

Sets of rat monoclonal antibodies directed to cell wall matrix polysaccharides/glycoproteins (Table 1) were used in immunolabelling procedures. Sections were incubated with 5% (w/v) milk protein in 1x PBS for 30 min. This solution was then replaced with primary antibody, diluted 1:5 in 5% milk protein/PBS. Slides were incubated for 2 h at room temperature. After 3 washes with 1x PBS the sections were incubated in secondary antibody (anti-rat IgG-whole molecule, FITC conjugate; Sigma Chemical Co.; 1:100 dilution in 1x PBS) for 1.5 h in the dark. An anti-mouse IgG–whole molecule FITC conjugate (Sigma Chemical) was used in experiments to detect mixed-linked glucan. Slides were washed three times with 1x PBS.

**Table 1:** The monoclonal primary antibodies used to investigate cell wall architectures.

Plant cell wall component		Antibody
Hemicellulose	Mixed Linkage Glucan	MLG^49^
	Xyloglucan	LM15^18^
	Mannan	LM21^50^
	Feruloylated xylan	LM12^51^
	Xylan	LM11^52^
Pectin	Galactan	LM5^53^
	Arabinan	LM6^54^
	Processed arabinan	LM16^46^
	DeSPHG	LM19^55^
	MPHG	LM20^55^
Glycoprotein	AGPs	LM2^56,57^

MPHG, Methyl Esterified Pectic Homogalacturonan; DeSPHG, De-esterified Pectic Homogalacturonan; AGPs, Arabinogalactan proteins.

#### Staining with Calcofluor White

Calcofluor White (Fluorescent Brightener 28, Sigma) diluted in PBS to 0.2 mg/ml, was added to the sections for 5 min in the dark. Slides were washed extensively with 1x PBS prior to application of antifade solution (Citifluor AF1; Agar Scientific) and a cover slip and then placed in the dark at 4 °C until examination.

#### Image analysis

Fluorescence imaging of Calcofluor white and FITC-conjugated antibodies was carried out using a Leitz DMRB fluorescence microscope, Leitz ultraviolet light source, QImaging-QIcam digital camera and Q-Capture Pro 6.0 software. A green fluorescence filter set (Semrock) with excitation wavelength of 472 ± 15 nm and emission wavelength of 520 ± 15 nm was used for visualisation of FITC. A filter set with excitation wavelength of 360 ± 20 nm and emission wavelength of greater than 425 nm was used for visualisation of calcofluor white. The captured images were analysed using Image-pro Analyser 7.0 (2009 Media Cybernetics). Representative micrographs were captured from a minimum of two biological (root segments) and two technical (sections from the same root) replicates for each condition and antibody. Commonly more than twice that number of replicates was analysed.

#### Pectate lyase treatment

Abundant pectic HG can mask other polysaccharides such as xyloglucan and mannan epitopes in plant cell walls^18^. Enzymatic degradation of HG was therefore carried out with pectate lyase prior to incubation with appropriate monoclonal antibodies. Sections were pre-treated with 0.1 M Na_2_CO_3_ for 2 h at room temperature in order to de-esterify the HG. Sections were then incubated in pectate lyase (recombinant, from *C*. *japonicus*; Megazyme) at 10 µg/ml in CAPS (N-cyclohexyl-3-aminopropanesulfonic acid) buffer for 2 h and then washed 3 times with purified water prior to immunolabelling as described above. The LM19 antibody was used to monitor the loss of HG.

### Nematode susceptibility of Arabidopsis cell wall mutants

Arabidopsis cell wall-related mutants: *bgal5* (β-*galactosidase 5*; SALK_139681), *msr1-2 (mannan synthesis related 1*; SALK_075245), *agp8 (arabinogalactan protein 8*; SALK_141852; expressed in roots^19^), *arad1 (arabinan deficient 1*; SAIL_905_E08^20^), *arad2 (arabinan deficient 2*; SALK_096544^21^), *qul1 (quasimodo2-like 1*; SALK_094635), *pme31 (pectin methylesterase 31*; SALK_074820; implicated in susceptibility to bacterial infection^22^) were obtained from The European Arabidopsis Stock Centre (Table 2 and Supplementary Table 1 for fuller details). The homozygosity of all mutants was confirmed by PCR using the gene-specific and T-DNA insert-specific primers indicated in Supplementary Table 1. Nine cm diameter pots were filled with soil mix (40% loam/ 40% sand/ 20% compost) and 5 seeds were sown in each pot. When the primary roots reached the bottom of the pots, 100 J2 *M*. *incognita* were introduced into the soil around the root system of each plant at a depth of approximately 50 mm (total of 500 J2s per pot). For each experiment 30 wild-type and 30 mutant plants were infected and each complete experiment was replicated on two separate occasions. At 21 dpi each root system was removed from the soil, carefully washed and stained with acid fuchsin as described previously^23^. The number of nematodes established in each root system was counted using a stereobinocular microscope (Olympus SZX9, Japan). The projected surface area of stained nematodes was measured from captured images using Image-pro Analyser 7.0 (Media Cybernetics) for 32 randomly selected nematodes from each set of wild-type or mutant plants. Statistical analysis of nematode size and number was performed using independent-samples Mann-Whitney U test (SPSS).

**Table 2:** Arabidopsis cell wall-related mutants used to investigate the role of cell wall components in root-knot nematode parasitism.

Plant cell wall component	Locus	Mutant name	Full name
Mannan	At3g21190	*msr1*	*mannan synthesis related 1*
Galactan	At1g45130	*bgal5*	*beta-galactosidase 5*
Arabinan	At2g35100	*arad1*	*arabinan deficient 1*
Arabinan	At5g44930	*arad2*	*arabinan deficient 2*
Arabinogalactan protein	At2g45470	*agp8*	*arabinogalactan protein 8*
Homogalacturonan	At1g13860	*qul1*	*quasimodo2 like 1*
Homogalacturonan	At3g29090	*pme31*	*pectin methylesterase 31*

## Results

**Figure 1:**
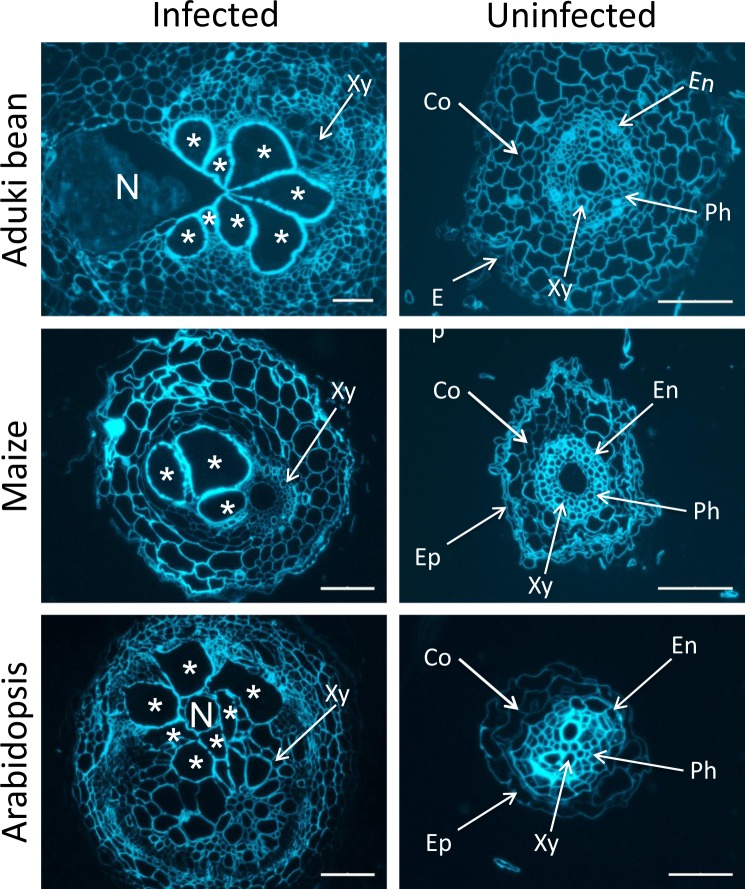
Comparative anatomies of root-knot nematode-infected and uninfected aduki bean, maize and Arabidopsis roots. Calcofluor-white-stained transverse sections. Asterisks indicate nematode-induced giant cells; N, indicates female *Meloidogyne incognita*; Co, cortical cells; Xy, xylem vessels; En, endodermis; Ep, epidermis; Ph, phloem cells. Scale bars represent 100 µm (infected sections) or 50 µm (uninfected sections).

Transverse sections of roots of Arabidopsis, aduki bean and maize that harboured *M*. *incognita* feeding sites at 21 days post infection (dpi), together with comparable sections of uninfected roots, were prepared for immunofluorescence analysis of cell wall matrix polysaccharides and glycoproteins. Calcofluor-white staining, allowing observation of anatomical features, confirmed that there was a striking enlargement of the vascular cylinder in the infected roots compared to uninfected roots in the three host species (Fig. 1). As the giant cells from which *M*. *incognita* feed are induced in the vascular cylinder, this study focuses particularly on the changes that occur in that region of the root.

### Cell wall architectures of the giant cells induced by RKN in three host species

**Figure 2:**
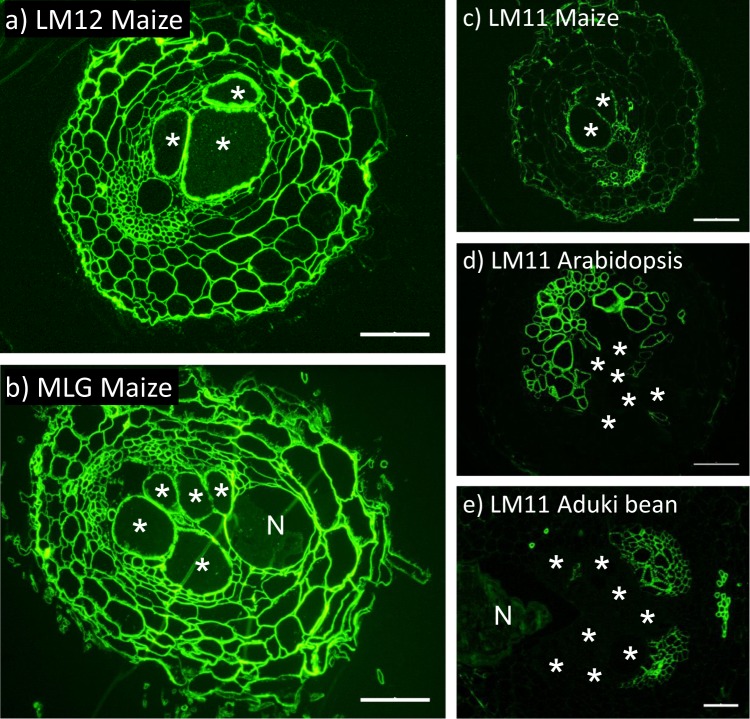
Immunolabelling of feruloylated xylan, mixed linkage glucan (MLG) and xylan in the roots of maize, Arabidopsis and aduki bean in nematode infected sections at 21 days post infection (dpi). The LM12 antibody binds feruloylated xylan only in the root sections of maize (**a**). The MLG antibody was used to localize mixed linkage glucan only in cell walls of maize (**b**). The LM11 antibody binds xylan in the root sections of maize (**c**), Arabidopsis (**d**) and aduki bean (**e**). Asterisks indicate giant cells in the nematode feeding site; N, nematode (*Meloidogyne incognita*); Bars: 100 µm.

In broad terms maize cell walls would be expected to have lower levels of pectic polysaccharides and xyloglucan than the dicotyledon aduki bean and Arabidopsis cell walls. In contrast, enhanced levels of heteroxylan with the commelinid monocotyledon feature of feruloylation would be expected, and the presence of mixed-linkage glucan (MLG) characteristic of the Poaceae. These differences were indeed reflected in the epitopes detected in the cell walls of Arabidopsis, aduki bean and maize all infected with the same root-knot nematode species, *M*. *incognita*. The LM11 xylan epitope, the LM12 ferulate epitope and the MLG epitope were detected in the giant cell walls only of maize roots (Fig. 2). The ferulate and MLG epitopes were absent from all cell walls of aduki bean and maize roots, whilst the LM11 xylan epitope was associated only with the proliferated xylem vessels of these parasitized roots (Fig. 2d,e).

**Figure 3:**
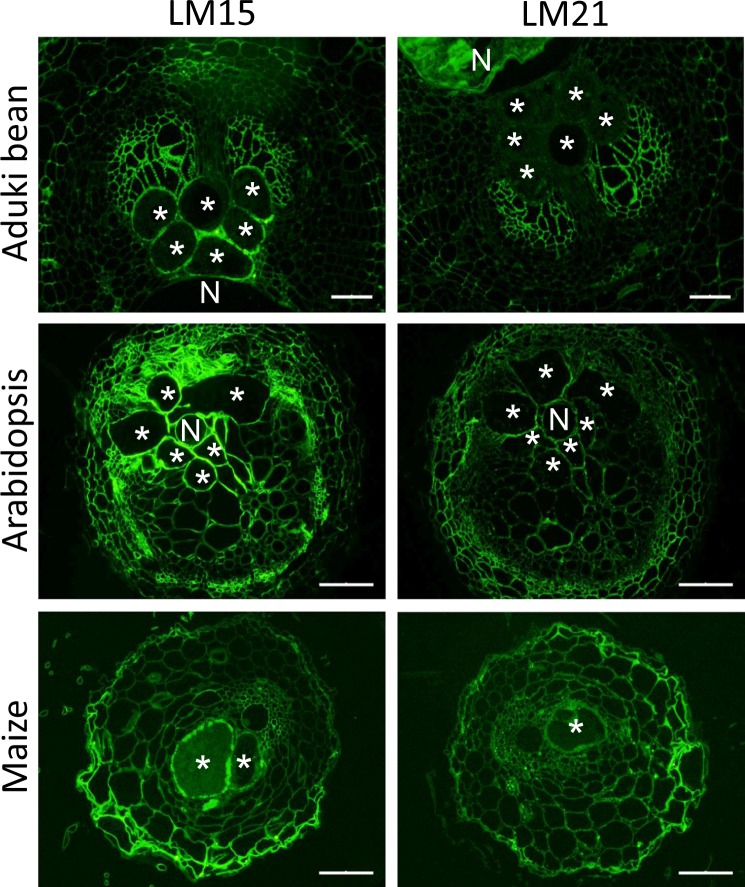
Immunolocalization of xyloglucan and mannan in infected root sections of Arabidopsis, aduki bean and maize at 21 days post infection with *Meloidogyne incognita*. The LM15 and LM21 antibodies localize xyloglucan and mannan, respectively. Asterisks represent giant cells; N, indicates the nematode. Bars: 100 µm.

Other non-cellulosic, non-pectic polysaccharides include xyloglucan and heteromannan. The xyloglucan epitope bound by LM15 was detected in all giant cell walls in all three host species with enzymatic removal of pectic HG being required for the strongest detection (Fig. 3). Detection of the LM21 mannan epitope was also optimized by the enzymatic removal of pectic HG. The LM21 epitope was detected in cell walls of giant cells of Arabidopsis and maize but not those of aduki bean (Fig. 3).

### Pectic polysaccharide and arabinogalactan-protein epitope modulations in response to RKN infection across three species

**Figure 4:**
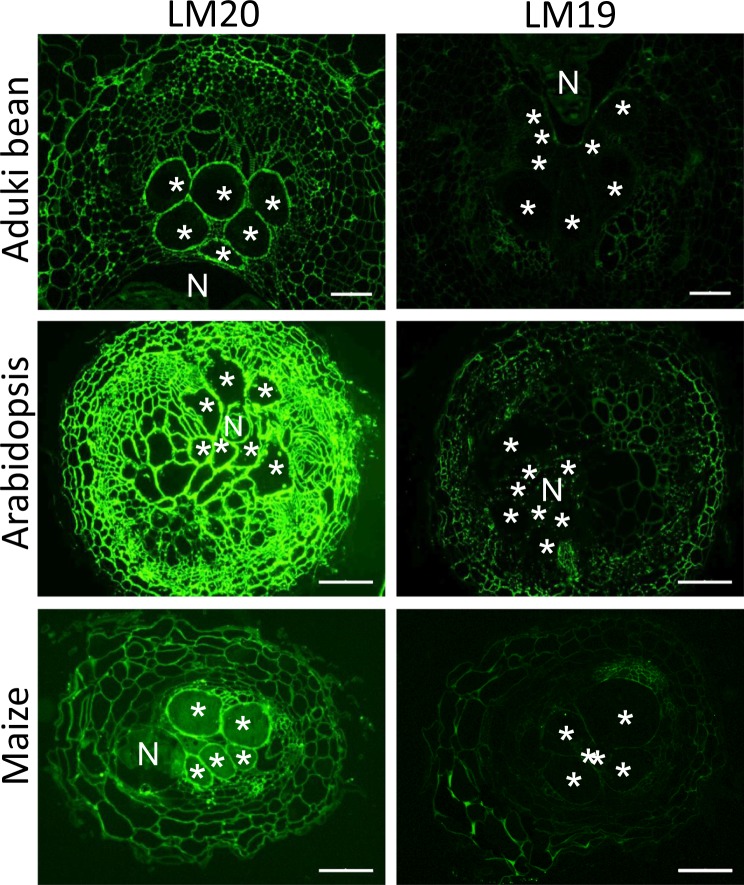
Immunolabelling of methyl esterified and de-esterified pectin homogalacturonan in nematode infected Arabidopsis, aduki bean and maize root sections at 21 days post infection with *Meloidogyne incognita*. The LM20 antibody recognises methyl esterified pectic homogalacturonan and the LM19 antibody recognises de-esterified pectic homogalacturonan. Asterisks indicate giant cells in the nematode feeding site; N, nematode; Bars: 100 µm.

**Figure 5:**
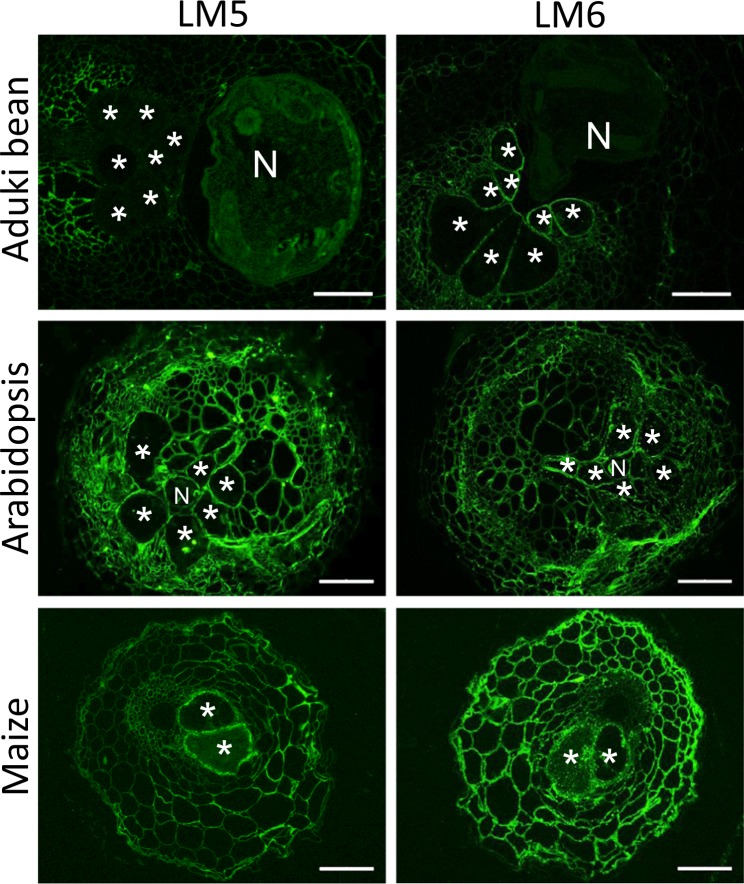
Immunolocalization of arabinan and galactan in infected root sections of Arabidopsis, aduki bean and maize at 21 days post infection with *Meloidogyne incognita*. The antibodies LM6 and LM5 bind arabinan and galactan, respectively. Asterisks represent giant cells; N, indicates the nematode. Bars: 100 µm.

Pectic HG in giant cell walls of all three species was highly methyl-esterified with strong detection of the LM20 epitope and weak detection of the LM19 epitope (Fig. 4). Rhamnogalacturonan-I is a highly heterogeneous set of pectic polysaccharides that appears to have the potential for hypermodulation of structure. This can be reflected in the presence of arabinan- and galactan-rich side chains that can be detected by LM6 and LM5 monoclonal antibodies respectively. In the case of RKN and the three plant species studied here the LM6 arabinan epitope was detected in all giant cell walls and for aduki bean this reflected a clear differential detection in comparison with surrounding cells where the detection was low (Fig. 5). The LM5 galactan epitope was detected in the giant cell walls of Arabidopsis and maize but not in those of aduki bean.

**Figure 6:**
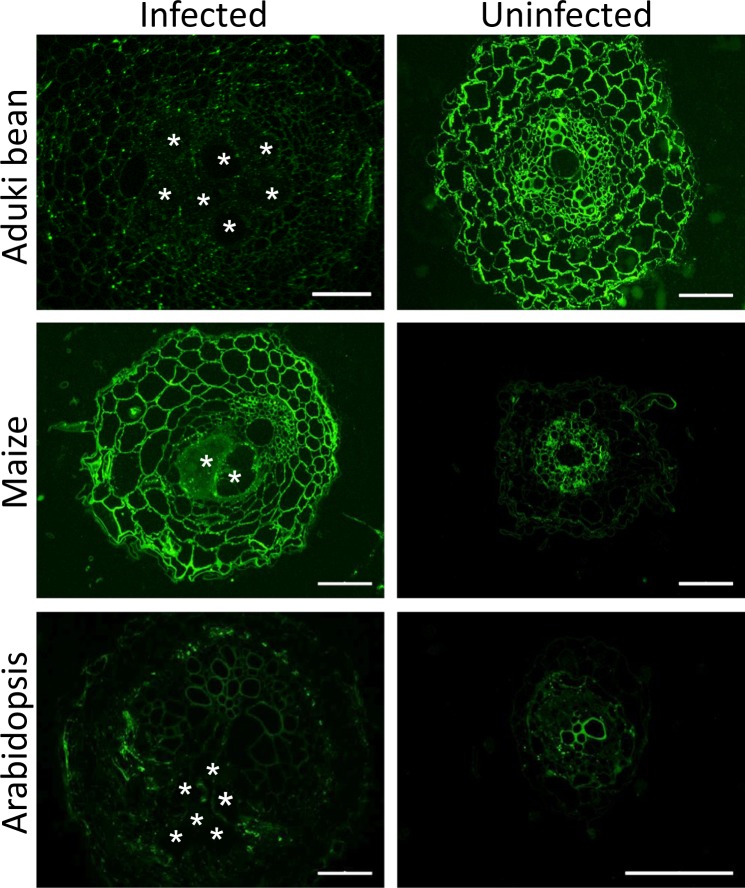
Immunolabelling of arabinogalactan proteins (AGPs) in nematode infected and uninfected Arabidopsis, aduki bean and maize root sections at 21 days post infection with *Meloidogyne incognita*. LM2 localised AGPs in nematode infected and uninfected root sections of host plants. N, represents the nematode; Bars: (infected sections) 100 µm; (uninfected sections) 50 µm.

The LM2 epitope is widespread and routinely used to detect AGPs. It was only weakly detected in association with giant cell walls in aduki bean and Arabidopsis and more strongly in those formed in maize (Fig. 6). A pattern of cross-species modulation of epitope detection in relation to RKN infection was observed for the LM2 AGP epitope across all root cell walls as shown in Fig. 6. This was particularly the case for aduki bean where the LM2 epitope was much more abundant in sections of uninfected roots.

### Impact of genetically modulated cell wall components on infection of *Arabidopsis* by *M*. *incognita*

**Figure 7:**
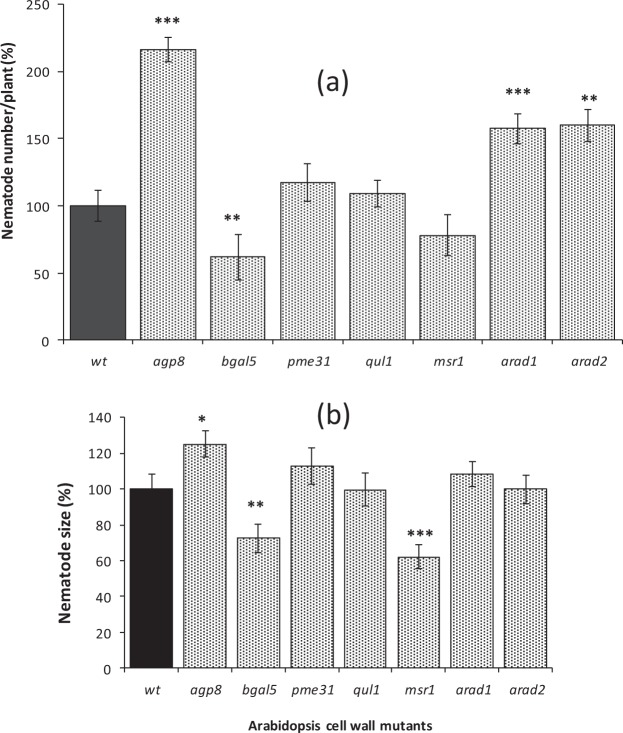
*Meloidogyne incognita* infection of Arabidopsis wild type (wt) and cell wall-related mutant plants. Mean number of nematodes per root system of wild type and mutant plants expressed as % relative to wild type (**a**). Mean nematode size in Arabidopsis wild type and mutant roots at 21 days post infection as % relative to wild type (**b**). Error bars represent standard error of the mean. For nematode number, n = 33–50 root systems from plants harvested on two separate occasions. For nematode size, n = 32 randomly selected individuals. Asterisks represent significance level ***P ≤ 0.001; **P ≤ 0.01; *P ≤ 0.05.

As an alternative approach to understand the functional importance of giant cell wall components, a set of relevant Arabidopsis mutants was used to explore their impact on nematode infection. Each T-DNA insertion line was mutant for a gene that influences one of the cell wall components analysed above. The impact of mutations was therefore on all cell walls and not specifically the walls of giant cells or gall regions, however no visible growth phenotypes were apparent for any of the mutant plants. Total nematode burden indicates success of root invasion, while size of the nematodes at the time point analysed reflects their rate of development and is therefore indicative of feeding site function and parasitic success. There was no significant difference in either the total number of nematodes per plant or the mean size of nematodes infecting roots of the pectic HG-related mutants *quasimodo2-like1* (*qul1*) and *pectin methylesterase 31* (*pme31*) relative to wild type (Fig. 7). However, analysis of the roots of these mutants revealed little difference in their cell wall composition relative to wild type plants: the pectic HG remained highly methyl-esterified with strong detection of the LM20 epitope and weaker detection of the LM19 epitope (Supplementary Figs 1 and 2). Roots of the *β-galactosidase 5* mutant (*bgal5*) harboured fewer nematodes than wild type plants and those nematodes were also significantly smaller. This correlates with the mutants having lower LM5 epitope abundance, indicative of galactan-rich side chains (Supplementary Fig. 3). The mannan synthesis-related mutant *msr1*, which had lower mannan (LM21) content compared to wild type (Supplementary Fig. 4), also harboured nematodes that were smaller, but their numbers were not significantly reduced (Fig. 7). In contrast, a knock-out of *arabinogalactan protein 8* (*agp8*) that caused a decrease in epitope recognition of LM2 and the *arabinan-deficient* mutants (*arad1* and *arad2*) that showed reduced binding of LM6 (Supplementary Figs 5 and 6), caused a significant increase in nematode numbers, relative to wild type (Fig. 7a). An increase in nematode size, however, was only observed for the *agp8* mutant (Fig. 7b).

## Discussion

The specific composition of the cell walls of nematode giant cells has not previously been defined and compared between hosts. Here, in three very different plant species – with varying cell wall matrix polysaccharide biochemistries - the same nematode has induced formation of giant cells whose walls have both common features and species-specific features.

### Commonalities of cell wall structures

The cell walls of giant cells induced by RKN require thickening, loosening to allow expansion, and the formation of ingrowths that increase the plasmalemma surface area to support increased solute uptake^24^. In previous work, abundant high-methylester HG, xyloglucan and arabinan were proposed to provide the flexible structure required for growth and maintenance of turgor pressure in the cell walls of the syncytial feeding site of a cyst nematode^25^. These three wall components are now shown also to be characteristic of *M*. *incognita* giant cell walls in three different host species. This is persuasive of high-methylester HG, xyloglucan and arabinan being important features of nematode feeding cells that facilitate their function in nutrient flow. When HGs are first incorporated into cell walls they are highly methylesterified and are later de-esterified by the action of pectin methylesterases (PMEs). This step is required for the formation of HG-calcium complexes, which are thought to induce pectic gel formation and thus cause cell wall stiffening^26,27^. Maintenance of the methylesterified state of HG in walls of giant cells may therefore contribute to wall flexibility required during nematode feeding. The abundant presence of arabinan may also be connected to a requirement for flexibility of giant cell walls. The high arabinan content of guard cell walls helps to maintain their flexibility during changes in cell volume and shape^28^.

It is likely that such cell wall alterations associated with expansion and function of the giant cells are mediated by induced regulation of host genes^2^. The genome of *M*. *incognita* is predicted to encode 81 cell wall degrading enzymes that can target pectin, arabinan, cellulose and xylan. In addition the nematode produces expansin-like proteins that may loosen cell walls and increase accessibility to those enzymes^29,30^. However many of these proteins, although secreted from the pharyngeal gland cells into the plant, are primarily involved in the parasitic interaction during early stages of infection and nematode movement through roots^31,32^.

Arabinogalactan proteins were detected in the walls of giant cells formed in all three host plant species with the strongest signal observed in maize. They have similarly been detected in the cell wall of the syncytial feeding sites of cyst nematodes in roots of Arabidopsis^24^ and potato, but not soybean^33^ Arabinogalactan proteins consist predominantly of galactose and arabinose components, as well as glucuronic acid, rhamnose and other monosaccharide residues together with a protein backbone particularly rich in hydroxyproline residues^34^. They are implicated in a wide range of plant processes including cell division, programmed cell death, secondary cell wall deposition, organ abscission and cell wall mechanics^35^ but the heterogeneous nature of AGP composition in roots^36^ makes it difficult to discern their function in giant cells.

### Specific changes in the walls of giant cells formed in aduki bean

Giant cell walls of aduki bean are notable, in comparison with Arabidopsis and maize, for two reasons and these are the absence of detectable galactan and mannan epitopes. Galactan-rich RG-I polysaccharides have been associated with increased cell wall firmness and reduced elasticity^37^. Our results show that detectable galactan is suppressed in the walls of giant cells induced in aduki bean; perhaps suggesting an additional factor to increase cell wall flexibility, highlighted above, as a possible important attribute of giant cell walls. The reported absence of the LM5 galactan epitope from cyst nematode syncytial cell walls in Arabidopsis^25^ could have comparable consequences for flexibility. Similarly, the mannan epitope (LM21) was detected in giant cell walls of maize and Arabidopsis but was suppressed within walls of giant cells in infected aduki bean. This may also be related to differences in cell wall mechanics although the role of the structurally diverse mannan polysaccharides is not well defined in cell walls beyond a possible role in tethering cellulose microfibrils and contributing to cell wall strength. The increased cell proliferation observed in the nematode feeding site around the giant cells of aduki bean compared to maize and Arabidopsis, suggests the response of aduki bean to *M*. *incognita* infection may be different from other hosts.

### Analysis of Arabidopsis mutants

In order to understand if the observed differences in giant cell wall composition had functional consequences on the development of the plant-nematode interaction, we used a range of Arabidopsis mutants that were compromised in particular cell wall components. Three broad categories of effect on nematode parasitism were detected: positive, negative or no apparent impact. Perhaps surprisingly, both of the mutants with no effect are related to the methylesterification status of homogalacturonan, the major constituent of pectin^38,39^. The high level of HG methyl esterification, detected with the LM20 antibody, was one of the most striking and consistent features of the walls of giant cells formed in all three hosts. Pectin methylesterases remove methylester groups from homogalacturonan^40^, therefore reduced local activity of these enzymes, or increased activity of their inhibitors^41^, could underlie this observation. However infection of neither the *pme31* pectin methylesterase mutant, nor the *qul1* mutant that lacks a predicted pectin methyltransferase, resulted in significant changes in nematode parasitism. This could be explained by the fact that epitope analysis with LM19 and LM20 revealed little or no change in the methylesterification status of HG in the cell walls of the two mutants compared with wild type plants. *PME31* is one of 66 pectin methylesterase genes in Arabidopsis^42^ and there may well be functional redundancy in the family, although loss of this single gene did increase susceptibility of *pme31* to the bacterial pathogen *Pseudomonas syringae*^22^. Similar redundancy may exist within the pectin methyltransferases that function within the Golgi apparatus to methylate HG prior to its secretion into the cell wall^26^. It is likely that complex temporal and spatial interactions between various PMEs and their inhibitors contribute to the regulation of HG methylesterification in giant cell walls.

The suppression of mannan and galactan epitopes in the walls of giant cells formed in aduki bean suggests that they are not essential for giant cell function. However, the two tested mutants with a negative impact on root-knot nematode development are associated with mannan synthesis and galactan metabolism. The *AtMSR1* gene (*mannan synthesis-related 1*) is expressed strongly in the vascular tissue of roots and glucomannan is reduced by approximately 40% in *msr1* mutant plants^43^. Nematodes parasitising roots of the *msr1* plants were significantly smaller than those developing in the corresponding wild type roots, although the number of nematodes was not altered. Both nematode number and size were reduced on *bgal5* mutant plants that lack a cell wall localised beta-galactosidase of the a1 sub-family^44^ expressed in roots and other tissues of wild type plants^45^.

Arabinan-related components are abundant in the giant cell walls formed in all three wild type hosts. Interestingly however, all mutant genotypes with increased susceptibility to root-knot nematodes are compromised in arabinan-related cell wall components. The two pectic arabinan-deficient mutants supported greater numbers of nematodes but with no impact on nematode development as determined by the size of the animal. The increased infection rate could possibly be connected to changes in epidermal cell walls in the elongation zone of the roots^21^ where root-knot nematode J2s invade. The subsequent unaltered rate of nematode development may reflect that these particular mutations did not impact the specific arabinan composition of the giant cell walls. Both the *arad1* and *arad2* mutants are associated with changes in binding of LM13, which recognises longer, unbranched regions of arabinans, but not of LM6^21^ that detects an abundant short chain 1,5-linked arabinan epitope^46^ in all giant cell walls. Knockout of the *AGP8* gene increased both nematode number and size. AGPs are abundant in both roots and root exudates and have been implicated in triggering wound-like responses^47^. *AGP* genes are up-regulated during the interaction between root-knot nematode and a resistant soybean cultivar^48^. Combined with the observed positive impact of the *agp8* mutation on parasitism of *Meloidogyne*, this suggests that certain AGPs may play a role in defence against root-knot nematodes that is distinct from their presence in giant cell walls.

## Conclusion

The cell walls of the root-knot nematode-induced giant cells undergo extensive architectural modification. We have infected three different plant species with the same nematode species and it is evident that the hosts share commonalities in terms of high-ester pectic HG, xyloglucan and pectic arabinan as components of giant cell walls. These features, which may influence cell wall flexibility, are also shared with the cell walls of the syncytial feeding sites induced by cyst nematodes in host roots. The two types of feeding site are formed by very different processes, therefore this conservation suggests that these are key attributes required for feeding site function. Other components of the giant cell walls reflect the primary cell wall matrix biochemistry of the host, for example xylan and MLG were detected in giant cell walls of maize but not of Arabidopsis or aduki bean. It appears that a functional feeding site can be created by modulating different existing cell wall polysaccharides of the host, whilst maintaining a core set of constituents. Perturbation of plant cell wall components through use of Arabidopsis mutants highlights their importance in nematode invasion and/or development.

## Electronic supplementary material

Supplementary figures tables and legends

## Data Availability

All data generated or analysed during this study are included in this published article (and its Supplementary Information files).
