# Supplementary material for: Host-specific signatures of the cell wall changes induced by the plant parasitic nematode, Meloidogyne incognita
Source: Sci Rep. 2018 Nov 23;8:17302. doi: 10.1038/s41598-018-35529-7 (PMC6251906; doi:10.1038/s41598-018-35529-7)
Supplement: Supplementary file 1 — Supplementary figures tables and legends [file 41598_2018_35529_MOESM1_ESM.pdf]

Host-specific signatures of the cell wall changes induced by the plant parasitic nematode, *Meloidogyne incognita*

*Refik Bozbuga, Catherine J. Lilley, J. Paul Knox, Peter E. Urwin**

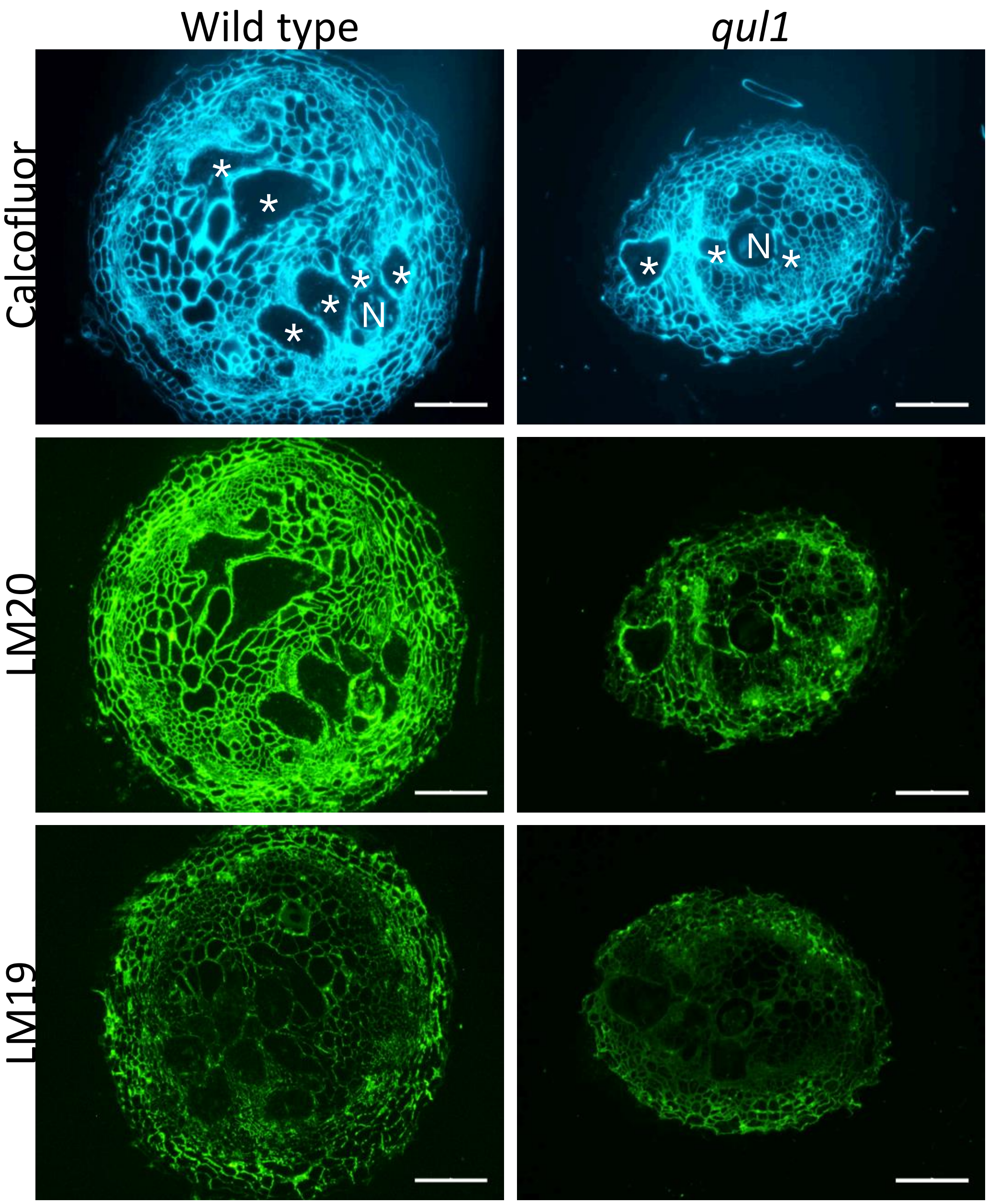

Supplementary Figure 1. Calcofluor-white-stained transverse sections and immunolabelling of methyl esterified and de-esterified pectin homogalacturonan in nematode infected Arabidopsis mutant *qul1* in comparison to infected wild type plants at 21 dpi. The LM20 antibody localizes the methyl esterified pectic homogalacturonan and the LM19 antibody localizes the de-esterified pectic homogalacturonan. Asterisks indicate giant cells in nematode feeding site; N, nematode; Bars: 100 µm.

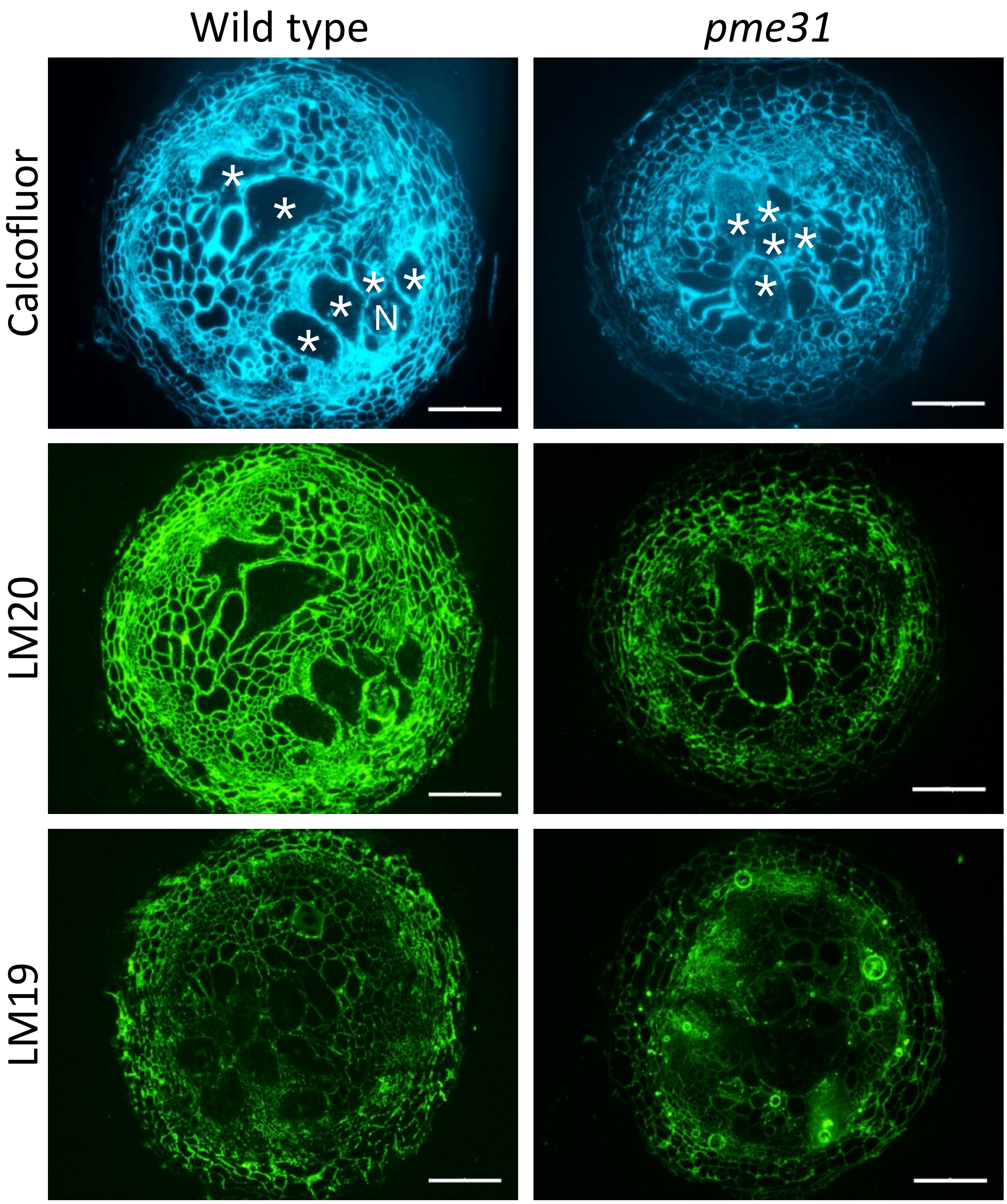

Supplementary Figure 2. Calcofluor-white-stained transverse sections and immunolabelling of methyl esterified and de-esterified pectin homogalacturonan in nematode infected Arabidopsis mutant *pme31* in comparison to infected wild type plants at 21 dpi. The LM20 antibody localizes the methyl esterified pectic homogalacturonan and the LM19 antibody localizes the de-esterified pectic homogalacturonan. Asterisks indicate giant cells in nematode feeding site; N, nematode; Bars: 100 µm.

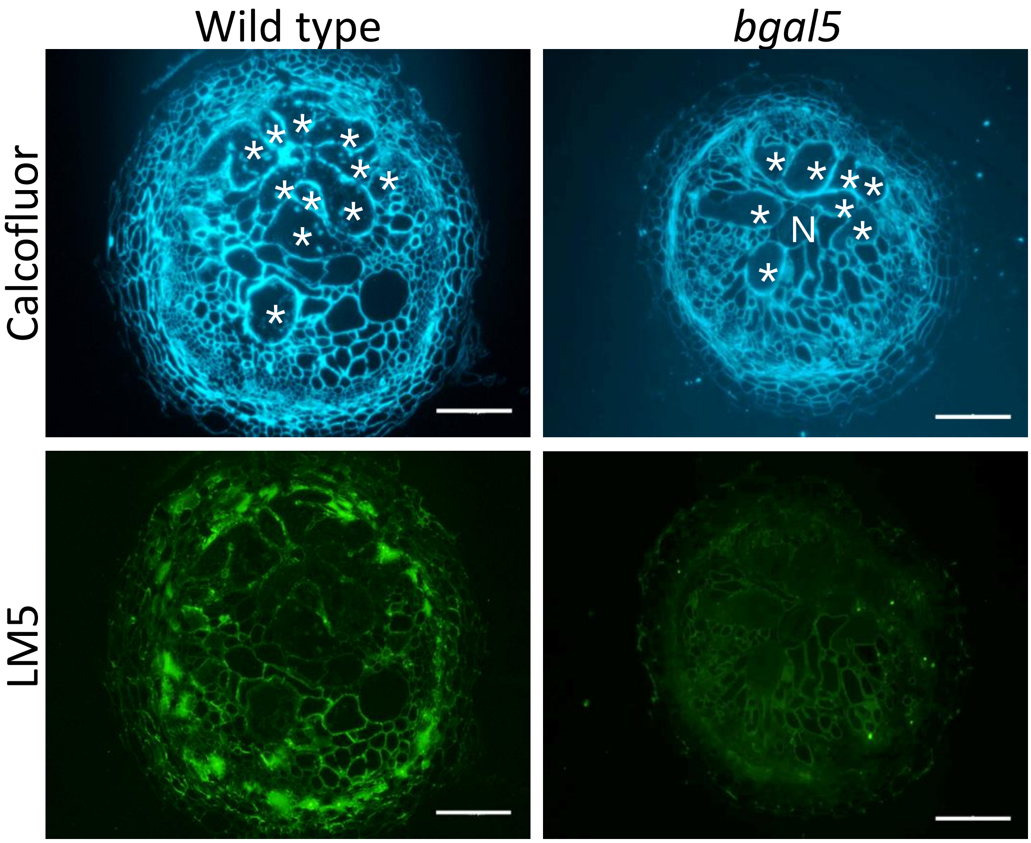

Supplementary Figure 3. Calcofluor-white-stained transverse sections and immunolabelling of galactan in nematode infected Arabidopsis mutant *bgal5* in comparison to infected wild type plants at 21 dpi. The LM5 antibody localises the galactan epitope, which is reduced in the mutant.. Asterisks indicate giant cells in nematode feeding site; N, nematode; Bars: 100 µm.

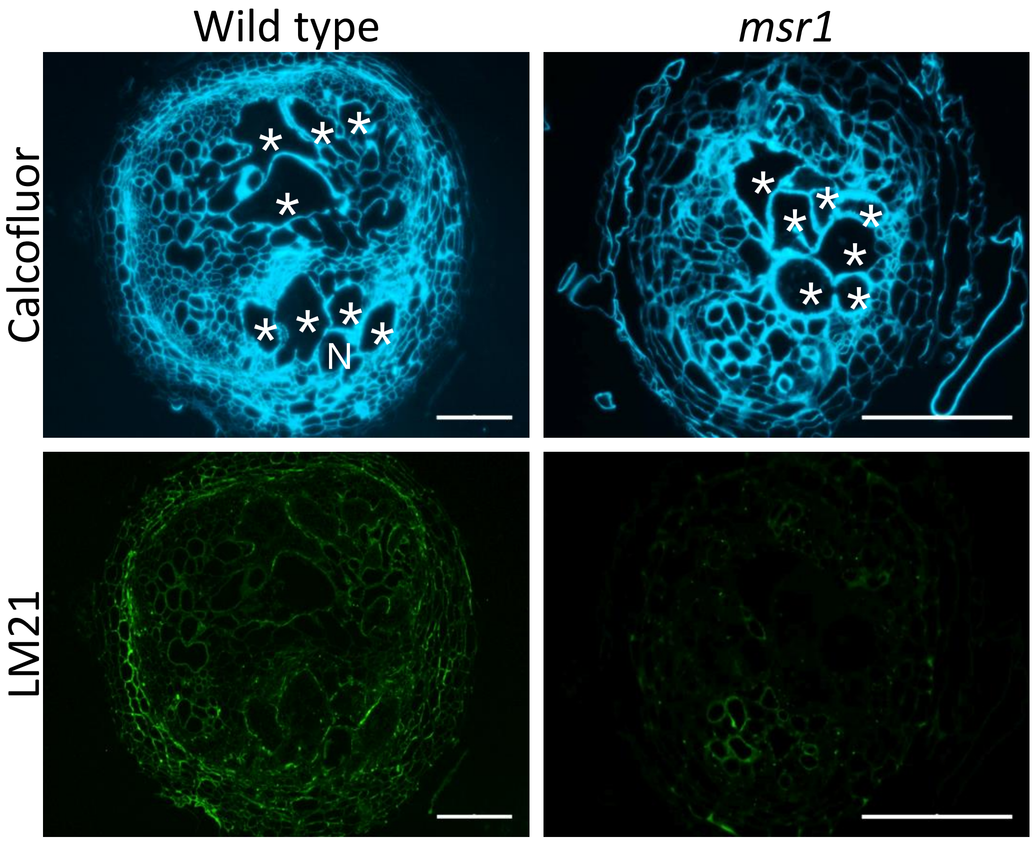

Supplementary Figure 4. Calcofluor-white-stained transverse sections and immunolabelling of mannan in nematode infected Arabidopsis mutant *msr1* in comparison to infected wild type plants at 21 dpi. The LM21 antibody localises the mannan epitope, which is reduced in the mutant. Asterisks indicate giant cells in nematode feeding site; N, nematode; Bars: 100 µm.

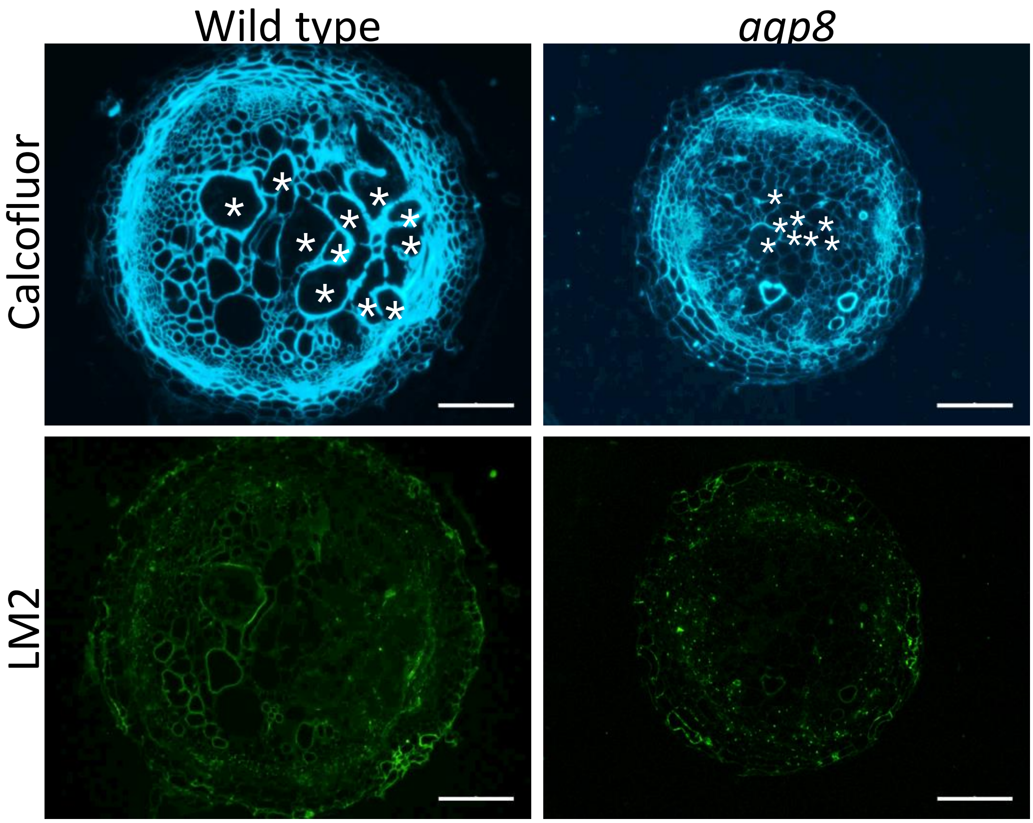

Supplementary Figure 5. Calcofluor-white-stained transverse sections and immunolabelling of arabinogalactan proteins in nematode infected Arabidopsis mutant *agp8* in comparison to infected wild type plants at 21 dpi. The LM2 antibody localises the arabinogalactan-protein epitope. Asterisks indicate giant cells in nematode feeding site; N, nematode; Bars: 100 µm.

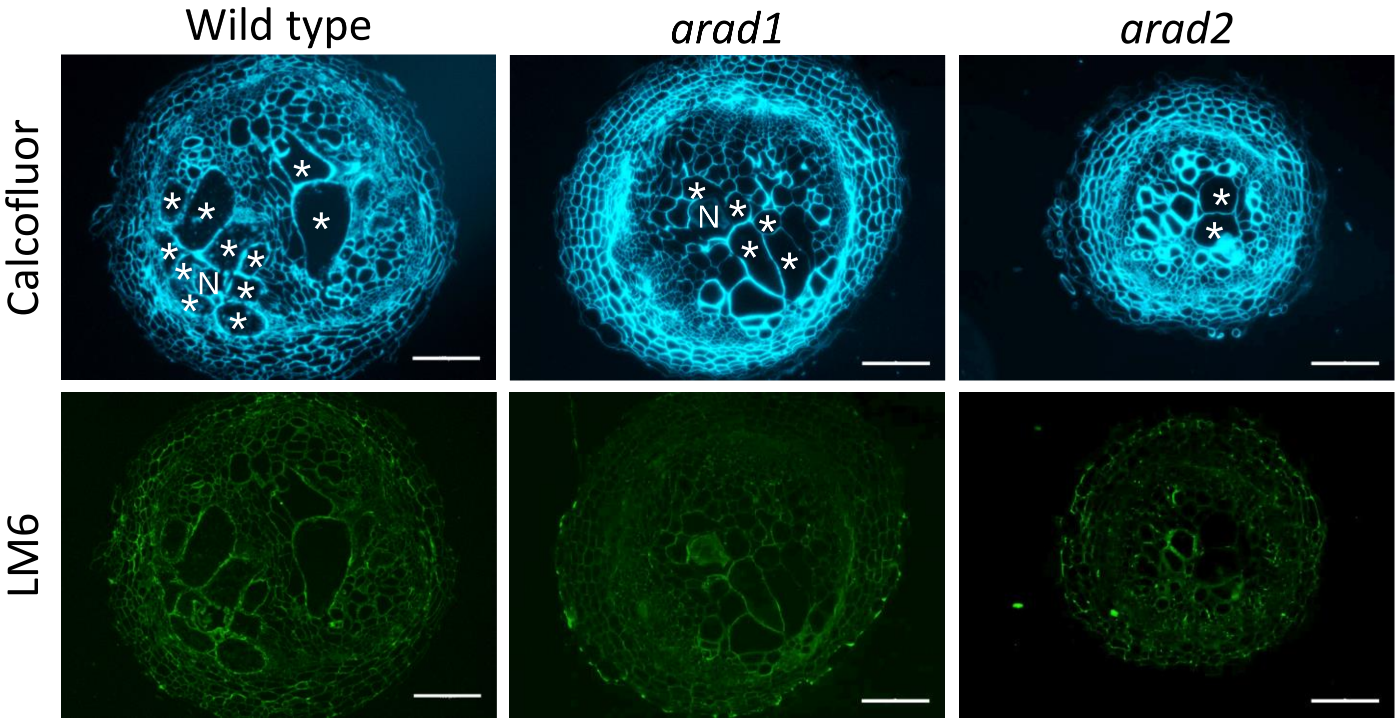

Supplementary Figure 6. Calcofluor-white-stained transverse sections and immunolabelling of arabinan in nematode infected Arabidopsis mutants *arad1 and arad2* in comparison to infected wild type plants at 21 dpi. The LM6 antibody localises the arabinan epitope. Asterisks indicate giant cells in nematode feeding site; N, nematode; Bars: 100 µm.

Supplementary Table 1: Primers for testing homozygosity of *Arabidopsis thaliana* cell wall mutants

**Gene locus**	**Gene name**	**Mutant ID**	**LP primer**	**RP primer**
At2g45470	*AGP8*	SALK_141852	ATGTAGAACATGAACGTCGGC	CTTTGCCTCCTTTAAGATCGG
At1g45130	*BGAL5*	SALK_139681	TGCTCCTATCGATGAATACGG	CTTAGAACTAACCGGCAACCC
At3g29090	*PME31*	SALK_074820	TCAAATTTCACCTAGGTGATTTG	CACAACCAAACGTACCAGTCC
At1g13860	*QUL1*	SALK_094635	CATTTGACATGGTCCACTGTG	TCATCACCCAAACTGATTTCC
At3g21190	*MSR1*	SALK_075245	CAAGACCTTCCATTTTTGGATC	TACAGGATCAGTTTCGCCATC
At2g35100	*ARAD1*	(Harholt *et al*., 2006)	TATGTGTTCAGGGTGGAAAAGT	GGGAGACTTGACGCCAGATT
At5g44930	*ARAD2*	(Harholt *et al*., 2012)	GTAGTTGTGTATACCCTAGACT	CGCCTCAGCCGGGTCAAAA

Harholt, J. *et al*. ARABINAN DEFICIENT 1 is a putative arabinosyltransferase involved in biosynthesis of pectic arabinan in Arabidopsis. *Plant Physiol*. **140,** 49-58 (2006).

Harholt, J. *et al.* ARAD proteins associated with pectic arabinan biosynthesis form complexes when transiently overexpressed in planta. *Planta* **236,** 115-128 (2012).
